# Características clínicas y angiográficas de pacientes con ectasia coronaria en un hospital de referencia

**DOI:** 10.47487/apcyccv.v3i3.229

**Published:** 2022-09-30

**Authors:** David Alejandro Rodríguez Falla, Eliana Alejandra Rafael-Horna, José Quiroz Burgos, Gerald Lévano-Pachas, Giovanni Meneses

**Affiliations:** 1 Departamento de Cardiología, Hospital Nacional Guillermo Almenara. Lima, Perú. Departamento de Cardiología, Hospital Nacional Guillermo Almenara Lima Perú; 2 Departamento Académico de Medicina Preventiva y Salud Pública, Facultad de Medicina, Universidad Nacional Mayor de San Marcos. Lima, Perú. Universidad Nacional Mayor de San Marcos Departamento Académico de Medicina Preventiva y Salud Pública, Facultad de Medicina, Universidad Nacional Mayor de San Marcos Lima Peru

**Keywords:** Ectasia, Angiografía Coronaria, Enfermedad Coronaria, Ectasia, Coronary Angiography, Coronary Disease

## Abstract

**Objetivo:**

: Analizar las características clínicas y angiográficas de pacientes con hallazgo de ectasia coronaria en la coronariografía.

**Materiales y métodos:**

Estudio descripitivo de pacientes admitidos a laboratorio de hemodinámica del Hospital Guillermo Almenara con hallazgo de ectasia coronaria, durante el periódo comprendido entre 2012 y 2020. Se determinó la frecuencia de ectasia coronaria, características clínicas, angiográficas y de flujo coronario*.*

**Resultados:**

se revisaron 7504 cateterismos y se halló 91 pacientes con ectasia coronaria (1,21%). De estos, 71 casos fueron varones (78%), y la edad media de 67,74 ± 9,9 años. El 38,5% de casos tuvieron obesidad o sobrepeso; 39,6 % fueron hipertensos; 11% diabéticos; 13,2% fumaban; 3,3% padecían enfermedad renal crónica y un 3,3% poliglobulia. El 61% de casos tuvieron diagnóstico de síndrome coronario agudo, y el 24 % de casos de angina estable de alto riesgo. La arteria comprometida por la ectasia con mayor frecuencia fue la coronaria derecha (70%). El diámetro promedio de la arteria ectásica fue de 5,7 mm. Se encontró trombo oclusivo en 19,8% de casos. Se halló asociación significativa entre el flujo TIMI y el diámetro de la arteria ectásica (p=0,000), además hubo asociación entre ectasia coronaria y síndrome coronario agudo entre los pacientes que residían a más de 2500 m de altitud (p=0,000).

**Conclusiones:**

la ectasia coronaria fue una entidad poco frecuente entre los pacientes sometidos a coronariografía, predominó en el sexo masculino, comprometió principalmente la coronaria derecha, se asoció a menor flujo TIMI, y a síndrome coronario agudo entre los residentes de más de 2500 m de altitud.

## Abstract (en)

**Objective:**

To analyze the clinical and angiographic characteristics of patients with coronary ectasia found on coronary angiography.

**Materials and methods:**

: Descriptive study of patients admitted to the cardiac catheterization laboratory of the Hospital Guillermo Almenara with coronary ectasia, during the years 2012 to 2020. The frequency of coronary ectasia, clinical, angiographic and coronary flow characteristics were determined.

**Results:**

7504 catheterizations were reviewed, and 91 patients were found to have coronary ectasia (1.21%). Of these patients, 71 cases were male (78%), and the mean age was 67.74 ± 9.9 years. The 38.5% of cases were obese or overweight; 39.6% were hypertensive; 11% diabetic; 13.2% smoked; 3.3% had chronic kidney disease and 3.3% had polyglobulia. Sixty-one percent of cases had a diagnosis of acute coronary syndrome, and 24% of cases had high-risk stable angina. The artery most frequently involved by ectasia was the right coronary artery (70%). The average diameter of the ectatic artery was 5.7 mm. Occlusive thrombus was found in 19.8% of cases. There was a significant association between TIMI flow and diameter of the ectatic artery (p=0.000), and there was also an association between coronary ectasia and acute coronary syndrome among patients living at an altitude of more than 2500 m (p=0.000).

**Conclusions:**

coronary ectasia was an infrequent entity among patients who underwent coronary angiography, was predominantly male, mainly involved the right coronary artery, was associated with lower TIMI flow, and acute coronary syndrome among residents above 2500 m of altitude.

## INTRODUCCIÓN

La ectasia coronaria fue descrita hace más de dos siglos por Bourgon, quien reportó en 1812 el hallazgo de dilatación de la arteria coronaria derecha en un estudio *post mortem*^1^^,^^2^. Packard reportó la primera revisión de doce casos ^3^, sin embargo, el término «ectasia» fue acuñado por Bjork (1966) en un reporte de tres casos de pacientes con tetralogía de Fallot a quienes se les realizó cateterismo cardiaco ^4^. La ectasia arterial coronaria es una entidad rara y es definida como una dilatación difusa de al menos 1,5 veces mayor del diámetro del vaso, con relación al segmento adyacente normal ^5^^-^^7^. Las tres arterias coronarias pueden estar afectadas, pero en alrededor de 75% de casos hay afectación uniarterial, y en pacientes con enfermedad coronaria isquémica concomitante los segmentos proximal y medio de la arteria coronaria derecha son los más comprometidos, seguidos de la arteria circunfleja y, por último, de arteria descendente anterior ^8^^-^^10^. Su prevalencia es variable entre 1,2 a 4,9%, siendo más frecuente en hombres que en mujeres (relación 3:1) ^(^^4^^,^^11^^,^^12^, se ha encontrado una incidencia mayor al 10% en pacientes con enfermedad coronaria isquémica concomitante ^13^; además, se ha reportado una tasa de mortalidad de 15% a los 7 años, la cual es similar a la tasa de mortalidad de enfermedad coronaria triarterial con tratamiento médico ^11^. 

Los mecanismos isquémicos no han sido del todo comprendidos; sin embargo, se acepta que es causado por disfunción microvascular; además, se ha reportado que el flujo lento y turbulento en los vasos dilatados resulta en trombosis e isquemia, llevando a embolismo de territorio distal del vaso ^14^; por otro lado, estos vasos ectásicos tienen tendencia al espasmo, con respuesta vasoconstrictora aumentada entre 65 a 93% en pruebas de ergonovina ^15^^,^^16^. Por lo cual, se ha postulado que las principales causas de isquemia son la alteración de perfusión debido al flujo lento y la consiguiente trombosis distal ^17^. 

La etiología puede comprender gran espectro de entidades clínicas tanto congénitas como adquiridas, incluyendo causas iatrogénicas, arteritis (poliarteritis nodosa, enfermedad de Takayasu, lupus eritematoso sistémico), síndrome de Marfan, émbolos sépticos infecciosos, enfermedad de Kawasaki y aterosclerosis, siendo esta última responsable de aproximadamente 50% de los casos ^18^. Se ha sugerido, también, que esta enfermedad representa una forma particular de remodelado arterial en respuesta a crecimiento de placa ateromatosa llamado «remodelamiento positivo», donde existe un remodelado expansivo excesivo, degradación enzimática de matriz extracelular, cambios en la media ^19^^-^^22^; e intervienen múltiples factores tales como: aumento de enzimas proteolíticas, metaloproteasas, homocisteína, hiperinsulinemia e incluso sobreestimulación crónica de óxido nítrico con el aumento consecuente de acetilcolina ^23^^,^^24^. La ectasia coronaria no tiene un tratamiento definido en sus distintas presentaciones clínicas, más allá del tratamiento establecido en el contexto de un síndrome coronario agudo.

El motivo del presente estudio es analizar las características clínicas y angiográficas de pacientes con ectasia coronaria, encontrados en el Hospital Guillermo Almenara Irigoyen (HNGAI) de Lima - Perú, durante el periodo comprendido entre febrero de 2012 y marzo de 2020.

## MATERIALES Y MÉTODOS

Estudio descriptivo, observacional, retrospectivo, de corte transversal. Se estudiaron pacientes adscritos al seguro EsSalud ingresados a sala de hemodinámica del Hospital Guillermo Almenara Irigoyen de Lima - Perú, durante el periodo febrero de 2012 a marzo de 2020. Se incluyeron a todos los pacientes con hallazgo angiográfico de ectasia coronaria durante el periodo.

Se definió ectasia coronaria como el diámetro ≥ 1,5 veces a segmentos adyacentes o del vaso normal, en uno o más vasos coronarios ^25^^,^^26^, y se revisaron todas las imágenes de los pacientes cuyos informes previos reportaron este hallazgo. La medición del calibre del vaso fue realizada por dos cardiólogos intervencionistas, siendo determinada según el diámetro del catéter utilizado; en caso hubiera discrepancia, se consultó a un tercer cardiólogo intervencionista. Se determinó las características clínicas de los pacientes, tales como: edad, sexo, factores de riesgo cardiovascular y antecedentes cardiovasculares. Se determinó la frecuencia de ectasia coronaria, y se clasificó de acuerdo con la propuesta por Markis *et al*. ^11^^,^^15^.

### Procedimientos

Se revisaron todas las películas de los pacientes con informe previo que reportaba ectasia coronaria. Dos cardiólogos intervencionistas del hospital evaluaron el flujo coronario mediante el TIMI y el TIMI Count Frame ^27^, con la correspondiente corrección de Gibson por arteria comprometida ^28^.

### Aspectos éticos

Los datos recolectados de los pacientes fueron tratados respetando el principio de confidencialidad. No se solicitó consentimiento informado ya que solo se revisaron y extrajeron datos de historias clínicas; los datos de las características del cateterismo cardiaco se evaluaron tras la hospitalización. 

### Procesamiento de datos

El procesamiento y análisis de resultados se realizó utilizando el *software* SPSS versión 25.0. Las variables fueron presentadas en tablas univariadas, bivariadas y gráficos de barras, lineales e histogramas. Se aplicó prueba de Kolmogorov y Smirnov para determinar la normalidad de las variables, resumidas con media y desviación estándar o mediana e intervalo intercuartilar, según el caso. Las variables cualitativas fueron asociadas mediante chi cuadrado o V de Cramer, mientras que en las cuantitativas se empleó r de Pearson o Rho de Spearman y análisis de varianza de una vía (ANOVA). Se conservó la confidencialidad de datos obtenidos en historia clínica mediante fichas de recolección, los cuales fueron de uso exclusivo para el presente estudio.

## RESULTADOS

**Figura 1:**
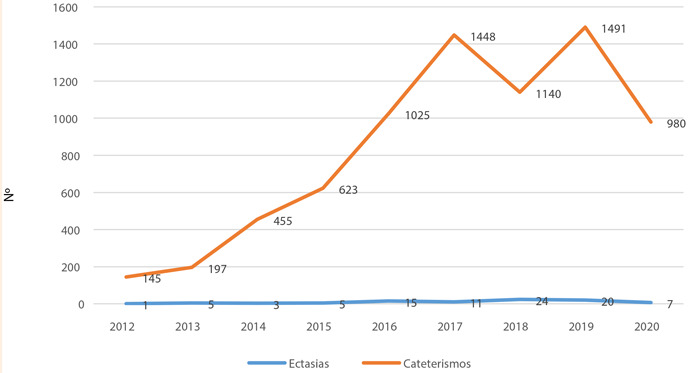
Total de cateterismos y casos de ectasia coronaria por año. HNGAI, 2012-2020

Durante los meses de febrero de 2012 a marzo de 2020 se realizaron 7504 cateterismos, se identificaron 91 pacientes con ectasia coronaria, por lo que la prevalencia lápsica de la ectasia coronaria encontrada en los cateterismos en dicho periodo fue de 1,21% (Figura 1).

**Figura 2:**
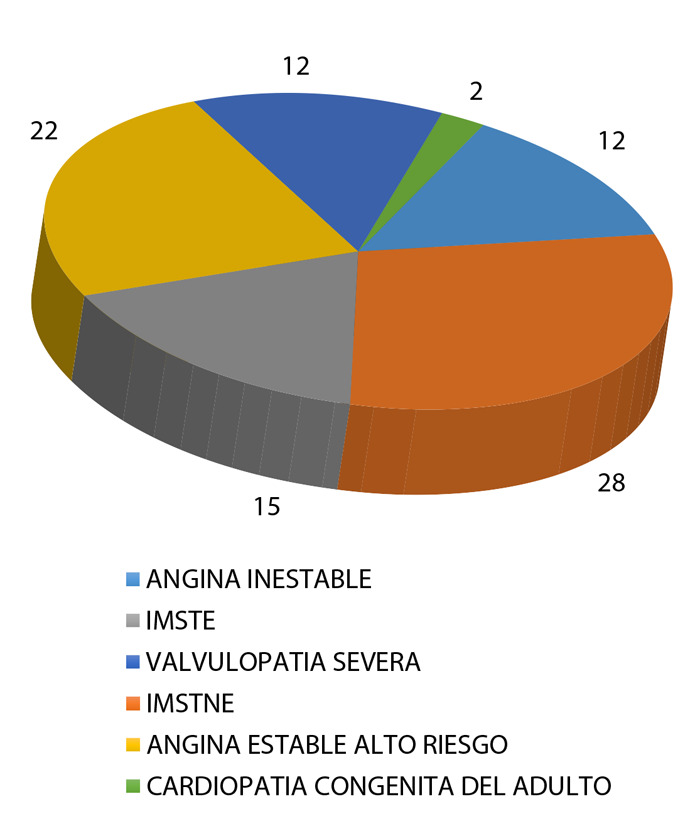
Diagnóstico de ingreso a sala de hemodinámica, en pacientes con presencia de ectasia coronaria

IMSTE: Infarto de miocardio con elevación del ST; IMSTNE: infarto de miocardio sin elevación del ST.

Setenta y un casos fueron de sexo masculino (78%) y 20 femenino (22%), con una media de edad de 67,74 ± 9,97 años. De ellos, 44% vivían a más de 2500 m de altitud sobre el nivel del mar. La obesidad o sobrepeso se encontró presente en 38,5% de los casos; 39,6% eran hipertensos; 11% diabéticos; 20,9% tenían cardiopatía isquémica crónica; 13,2% fumaban; 3,3% padecían enfermedad renal crónica y 3,3% poliglobulia (Tabla 1). Con respecto a los diagnósticos en el momento de la coronariografía, 61% tuvieron síndrome coronario aguda (SICA), y 24 % presentó angina estable de alto riesgo; además, 15% presentaban diagnóstico de valvulopatía severa o cardiopatía congénita del adulto (Figura 2). 

**Tabla 1:** Características clínicas de pacientes con ectasia coronaria.

Variable	N (%)
Edad	67,74 ± 9,97 *
Sexo masculino	71 (78,0)
Altitud de residencia	
<2500 m	51 (56,0)
>= 2500 m	40 (44)
Índice de masa corporal (kg/m^2^)	
<25	56 (61,5)
25-30	16 (17,6)
>30	19 (20,9)
Hipertensión arterial	36 (39,6)
Diabetes *mellitus*	10 (11,0)
Cardiopatía isquémica crónica	19 (20,9)
Tabaquismo	12 (13,2)
Enfermedad renal crónica	3 (3,3)
Poliglobulia	3 (3,3)

*Media +/- desviación estándar.

**Figura 3:**
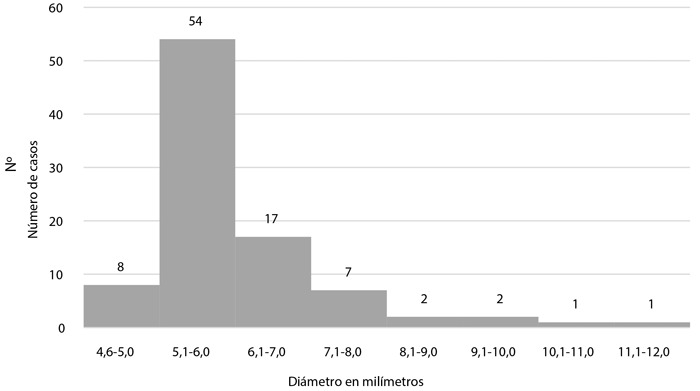
Distribución de pacientes con ectasia coronaria, según el diámetro en milímetros del vaso afectado

**Figura 4:**
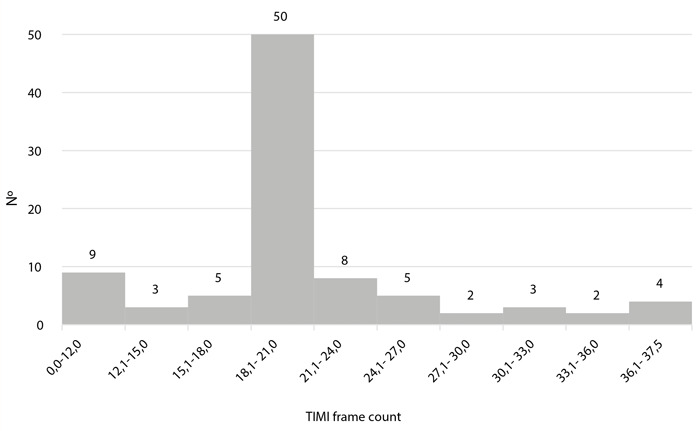
Distribución de pacientes con ectasia coronaria, según el TIMI *frame count* del vaso afectado

En cuanto a la arteria ectásica y la clasificación de ectasia, se observó que más de 70% correspondió a la coronaria derecha, seguida de la arteria circunfleja y descendente anterior, y más de la mitad de los casos de ectasia fueron de clase 3 (Tabla 2). El diámetro del vaso ectásico tuvo una mediana de 5,7 mm (RIQ: 5,30 - 6,40) (Figura 3) con presencia de trombo oclusivo en el 19,8% de los pacientes. Se diagnosticó enfermedad coronaria en 84,6% de pacientes, siendo el flujo TIMI predominante el de grado 3 (71,4%), seguido por el grado TIMI 2 (16,5%), TIMI 0 (7,7%) y TIMI 1 (4,4%). El *TIMI count frame* tuvo una mediana de 20,0 (intervalo intercuartil 18,9 - 21,1) (Figura 4).

**Tabla 2:** Distribución de pacientes con ectasia coronaria, según arteria comprometida y clasificación de Markis

	Clasificación de ectasia				Total
	1	2	3	4		
Nº de casos con arteria ectásica					
CD	20	0	36	10	66 (72,5)
CX	4	1	6	3	14(15,4)
DA	3	0	4	4	11(12,1)
Total n (%)	27 (29,7)	1 (1,1)	46 (50,5)	17(18,7)	91 (100)

CD: coronaria derecha. CX: circunfleja. DA: descendente anterior.

No se encontró asociación entre la clasificación de ectasia y sexo (p=0,156); cardiopatía isquémica crónica (p=0,106); diámetro del vaso (p=0,451); índice de masa corporal (p=0,173); hipertensión arterial (p=0,654); diabetes *mellitus* (p=0,229); tabaquismo (p=0,867), ni enfermedad renal crónica (p=0,853). En los pacientes de edad mayor/igual de 65 años hubo predominancia de la clase 3 de ectasia coronaria (52,7%) seguida de las clases 4 y 1 (25,5 y 21,8%, respectivamente); mientras que los menores de 65 años los porcentajes de las clases 3 y 1 fueron similares (47,2 y 41,7%, respectivamente). Se halló asociación significativa entre el flujo TIMI de la arteria comprometida y el diámetro del vaso (p=0,000), y correlación negativa estadísticamente signifiticativa entre el flujo TIMI del vaso con la edad del paciente y el índice de masa corporal (r= -0,232, p=0,027). Finalmente, encontramos una mayor frecuencia de ectasia coronaria y síndrome coronario agudo entre los pacientes que residían a más de 2500 m de altitud (p=0,000). 

## DISCUSIÓN

La prevalencia lápsica de ectasia encontrada en nuestra casuística fue de 1,21%, hallazgos que son similares a los descritos por Hartnell *et al*., Swanton *et al.*, Malviya *et al.*, y Makris *et al*. ^6^^,^^8^^,^^11^^,^^29^. La predominancia fue de los varones de 4:1, siendo semejante a lo reportado por Malviya *et al.*^29^, pero esta fue mayor en el trabajo de Hartnell *et al.*^5^, quienes hallaron una razón de 34:1, así como en el trabajo de Swanton *et al.*^8^, que de 12 pacientes identificados todos fueron varones; asimismo, en el trabajo de Bermudez *et al*. ^12^ quienes encontraron que 91,2% de sus pacientes fueron varones. La media de la edad fue de 67,7 años, mayor a lo reportado en las series de Swanton *et al*., Malviya *et al*. y Befeler *et al*., en donde las medias fueron de 53,08; 53,4 y 51 años ^6^^,^^8^^,^^29^, respectivamente; sin embargo, fue parecida a la serie de Bermudez *et al*. ^11^, quien encontró una media de edad de 63,3 años en pacientes con ectasia.

Se encontró afectación uniarterial en 50,5% de casos lo que demuestra que el tipo 3 de ectasia es el predominante. Hallazgos similares a los encontrados por Daoud *et al*. y Al Harthi *et al*. ^9^^-^^10^, el principal vaso afectado fue la coronaria derecha en 72,5%, seguido de la circunfleja y la descendente anterior, semejante a lo reportado por Befeler *et al*. en cuyos resultados los porcentajes de afectación de la circunfleja y descendente anterior fueron superiores ^6^. En la serie de Swanton *et al*. encontraron afectación similar de la coronaria derecha y la circunfleja, y solo un caso en la descendente anterior ^8^; Hartnell *et al*. también encontraron predominio de la coronaria derecha, pero en un porcentaje menor (40%) y el segundo vaso afectado fue la descendente anterior (29%) ^5^; por el contrario, en los resultados de Malviya *et al*. la arteria descendente anterior (59,6%) fue el vaso más comúnmente afectado, seguida de la arteria coronaria derecha (46,1%), y la circunfleja (36,5%) ^29^.

Se encontró coexistencia de enfermedad coronaria en una cifra muy superior a la hallada por Gunes *et al*., quienes encontraron asociación con enfermedad coronaria en 59% de pacientes ^30^; sin embargo, muy parecido a las series de Harikrishnan *et al*. y Lam *et a*l. quienes encontraron enfermedad coronaria en 84,7 y 82% de los casos, respectivamente ^31^^-^^32^. Alrededor del 60% de los casos se presentaron en el contexto de un síndrome coronario agudo, de manera similar a los resultados de Swanton *et al*. ^8^, mientras que Befeler *et al*. solo lo reportaron en el 25% ^6^^),^ Hartnell *et al*. en el 17,1% ^5^, y Malviya *et al*. apenas en 7,7% ^(^^29^^)^ de los casos. Befeler *et al*. encontraron el 100% de sus casos en el contexto de cardiopatía isquemica crónica, mientras que nosotros solo consignamos dicho hallazgo en 24%, en contraparte Malviya *et al*., lo registraron en 17,3% de sus casos ^6^^,^^29^.

Entre las limitaciones del trabajo destacamos que, al tratarse de un estudio unicéntrico, sus resultados no pueden representar a otras poblaciones. Sin embargo, debe tenerse en cuenta que nuestro hospital es un centro de referencia nacional, de modo que puede reflejar indirectamente la realidad de los usuarios de la seguridad social a nivel nacional. Por otro lado, existe un probable subregistro de antecedentes de los pacientes reportados en las historias clínicas. Debido a las características de la población y al no disponer de datos del resto de los pacientes sometidos a cateterismos, no ha sido posible realizar un análisis multivariado.

En conclusión, encontramos que la prevalencia de pacientes con ectasia coronaria es muy baja, predomina en el sexo masculino, la hipertensión arterial es el principal factor de riesgo encontrado, compromete principalmente de la coronaria derecha, y asoció con isquemia coronaria aguda entre los residentes de más de 2500 m de altitud.

Se recomienda comparar en posteriores estudios los resultados de los pacientes con ectasia frente al total de pacientes sometidos a coronariografía, para poder estudiar factores de riesgo o protectores, o un estudio prospectivo múlticéntrico sobre el manejo de estos pacientes y sus resultados a largo plazo.
